# New observations on maternal age effect on germline *de novo* mutations

**DOI:** 10.1038/ncomms10486

**Published:** 2016-01-19

**Authors:** Wendy S. W. Wong, Benjamin D. Solomon, Dale L. Bodian, Prachi Kothiyal, Greg Eley, Kathi C. Huddleston, Robin Baker, Dzung C. Thach, Ramaswamy K. Iyer, Joseph G. Vockley, John E. Niederhuber

**Affiliations:** 1 Inova Translational Medicine Institute, Inova Health System, 3300 Gallows Road, Falls Church, Virginia 22042, USA; 2 Department of Pediatrics, Virginia Commonwealth University School of Medicine, 1201 E Marshall St, Richmond, Virginia 23298, USA; 3 Inova Children's Hospital, Inova Health System, 3300 Gallows Road, Falls Church, Virginia 22042, USA; 4 Fairfax Neonatal Associates, Inova Health Systems, 3300 Gallows Road, Falls Church, Virginia 22042, USA; 5 Department of Obstetrics and Gynecology, Virginia Commonwealth University School of Medicine, 1201 E Marshall St, Richmond, Virginia 23298, USA; 6 Johns Hopkins University School of Medicine, 733 North Broadway Street, Baltimore, Maryland 21205, USA

## Abstract

Germline mutations are the source of evolution and contribute substantially to many health-related processes. Here we use whole-genome deep sequencing data from 693 parents–offspring trios to examine the *de novo* point mutations (DNMs) in the offspring. Our estimate for the mutation rate per base pair per generation is 1.05 × 10^−8^, well within the range of previous studies. We show that maternal age has a small but significant correlation with the total number of DNMs in the offspring after controlling for paternal age (0.51 additional mutations per year, 95% CI: 0.29, 0.73), which was not detectable in the smaller and younger parental cohorts of earlier studies. Furthermore, while the total number of DNMs increases at a constant rate for paternal age, the contribution from the mother increases at an accelerated rate with age.These observations have implications related to the incidence of *de novo* mutations relating to maternal age.

## Abstract (web summary)

The study of germline mutations has been greatly enhanced by massive parallel sequencing technologies. Here the authors use deep sequencing data from nearly 700 parent-child trios to show maternal age has a small but significant correlation with the number of *de novo* mutations in the offspring.

## Graphical abstract

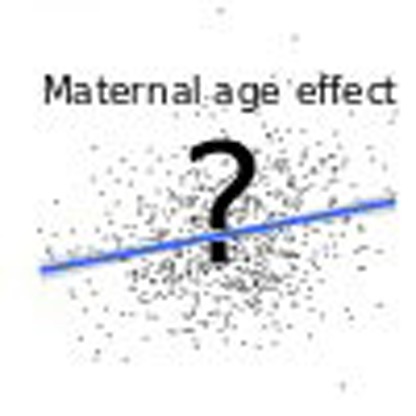

There have been a number of scientific reports supporting a positive correlation between paternal age and the frequency of germline *de novo* point mutations (DNMs) in the offspring. This correlation is especially evident in sporadic diseases that have been shown to be more frequent in the children of fathers of advanced paternal age^1^. The increase in the number of mutations has been hypothesized to be due to the accumulated number of cell divisions during spermatogenesis as the male ages. Several recent whole-genome (WGS) and whole-exome sequencing (WES) studies have used directly observed sequence data to confirm the effect of paternal age; for example, Kong *et al.* studied deeply sequenced whole-genome trio data obtained from 78 Icelandic families and showed a very strong correlation between the number of DNMs in the offspring and paternal age^2^. A trio-based WGS study of 250 Dutch families found a similar trend^3^.

To study the patterns of DNMs in a larger cohort of family trios, we developed a rigorous data analysis pipeline for identifying germline *de novo* single nucleotide mutations. The results reported here used both sequencing information (base and mapping qualities) and population genetic variation information obtained from public and our own cohorts. The deeply sequenced (∼60X) WGS data generated using the Complete Genomics Inc., (Mountain View, CA, (CGI)) platform was obtained from a large ethnically and racially diverse family-based case–control study on the genomic and clinical causes of preterm birth. Sixty-one of these trios were also sent to Illumina Inc., (La Jolla, CA) for whole-genome deep sequencing, assembly and variant calling.

In this study, we show that maternal age has a small but significant correlation with the total number of DNMs in the offspring, after accounting for paternal age. Interestingly, there is evidence that the mutation rate of the mothers increases at an accelerated rate with age, while the mutation rate of the fathers increases at a constant rate. The maternal age is also shown to be positively correlated with the number of DNMs of determined maternal origin using the Illumina sequencing data on 61 trios in the same cohort, providing further evidence for the potential maternal age effect on an orthogonal platform.

## Results

### Parents' ages and number of *de novo* mutations

Using a specifically developed analysis pipeline, we identified 28,230 DNMs from 719 mother–father–newborn trios (Supplementary Fig. 1 and Supplementary Table 1). We first fit a multiple linear regression (MLR) model to the data, regressing the number of DNMs on both the father's and mother's ages at conception, mode of pregnancy (whether involving assisted reproductive techniques (ART)), and the newborn's preterm status (gestational age <37 or otherwise). By the Bonferroni Outlier Test^4^, one sample with Bonferroni corrected *P* values <0.05 was removed before the model was refit. Both parental ages were shown to be significant determinants of the number of DNMs in the newborn (*P*<2.0 × 10^−16^ for father's age and *P*=4.28 × 10^−6^ for mother's age). The newborn's preterm status did not have a significant correlation with the number of DNMs (*P*=0.55) when controlling for other variables. The usage of ART was moderately significant (*P*=3.86 × 10^−3^), with 4.25 more DNMs on average compared with natural conception when controlling for other variables.

**Figure 1:**
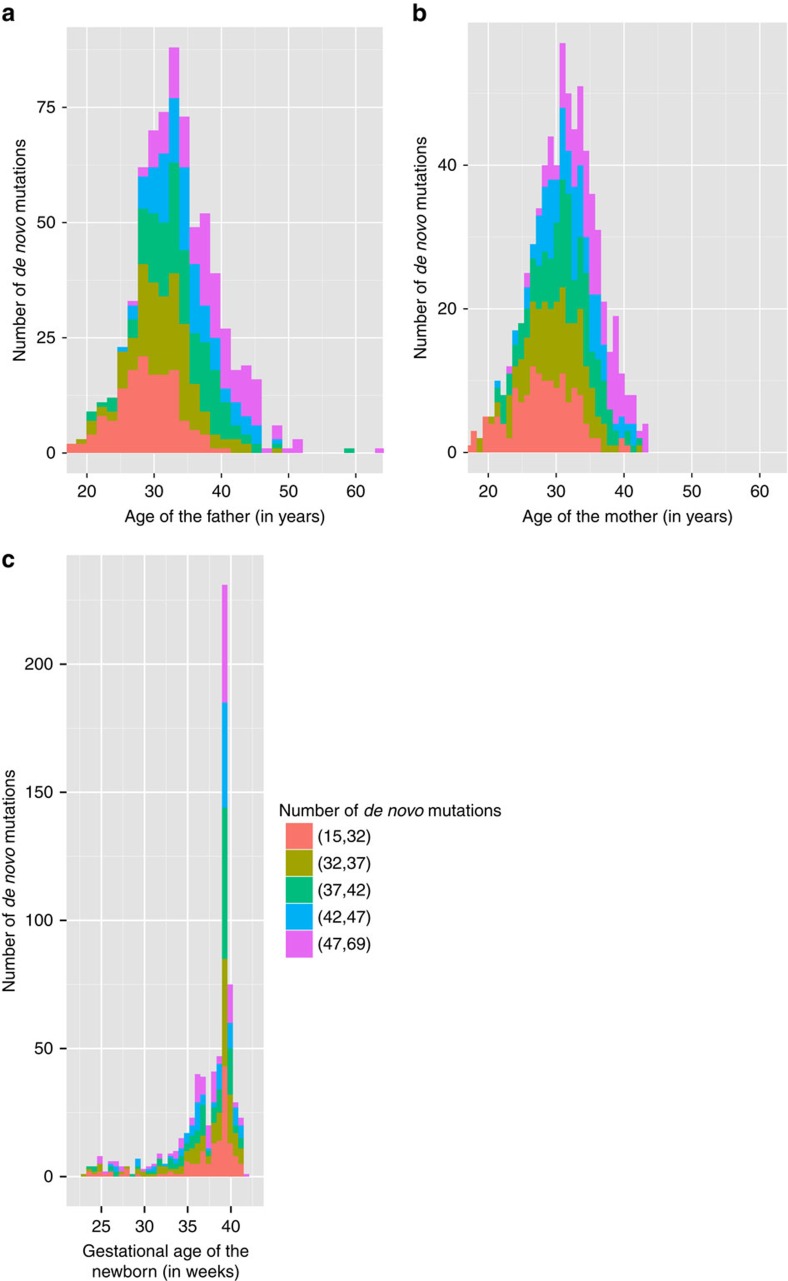
Parents' age distribution with number of *de novo* mutations in their offsprings. (**a**) The distribution of father's ages at conception (in years). (**b**) The distribution of mother's ages at conception (in years). (**c**) The distribution of gestational age for the newborns (in weeks). The colours in each bar in the histograms indicate the proportion of newborns with each number of DNMs in each of the five equally sized bins.

To focus on the effect of parental age, we removed the 25 trios with conceptions involving ART from our analysis. In the final set of 693 trios, the ages of the fathers at conception ranged from 17 to 63 years, with a mean of 33.4 years. Twenty-four (3.5%) of the fathers were older than 45 years, extending by 18 years the age range for the statistical model from previously reported whole-genome studies^2^,^3^. The ages of the mothers ranged from 17 to 43 years with a mean of 31.2 years. The gestational age of the newborns ranged from 23 to 42 weeks with a mean of 37.2 weeks (Fig. 1). The resulting data include a total of 26,939 DNMs, which is on average 38.87 DNMs per proband (s.d.=8.77, range=15–69 DNMs, Supplementary Data 1). Note that all the estimates given in this section are raw estimates without accounting for sensitivity, specificity and the callable genome sizes.

**Figure 2:**
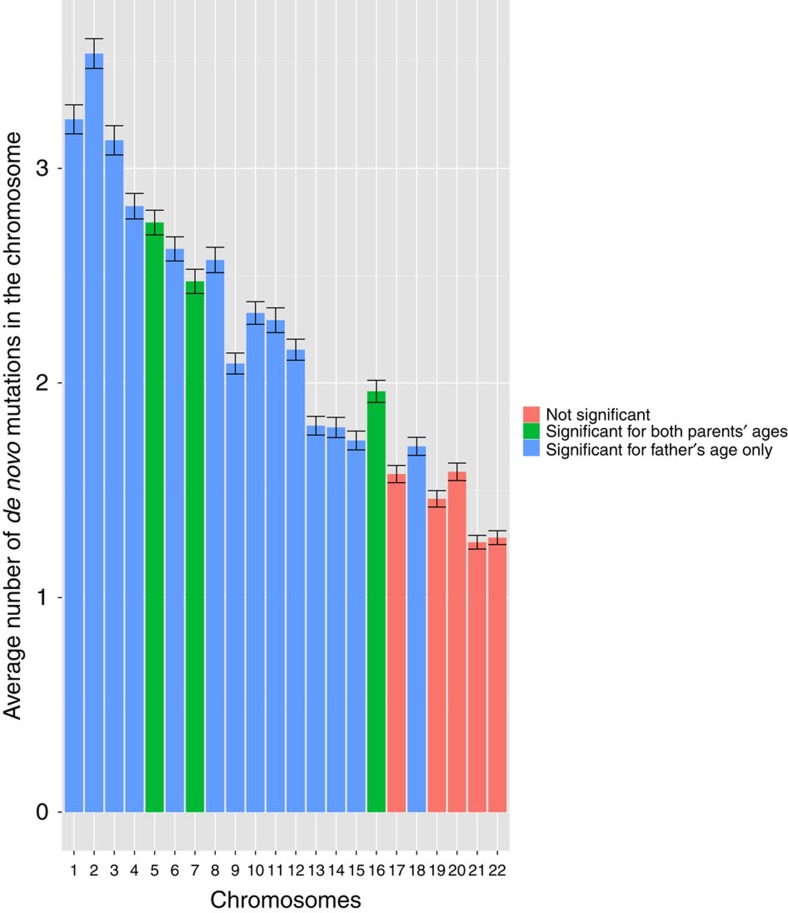
Bar plot of average number of de novo mutations by chromosome. The average number of DNMs in each autosome are largely correlated with the chromosome sizes. The error bars represent the s.e. of the mean DNMs for each chromosome. Numbers of DNMs in chromosomes 17, 19, 20, 21 and 22 are not significant for either father's or mother's age (*P*>0.05). Numbers of DNMs in chromosomes 1–4, 6, 8–15 and 18 are significantly correlated with father's age only. Numbers of DNMs in chromosomes 5, 7, 16 are significantly correlated with both parents' ages.

As expected, the total number of DNMs in the offspring is significantly correlated with the father's age (*P*<2.00 × 10^−16^, MLR) after controlling for mother's age, with the estimated rate of increase 0.64 DNMs per year of age (95% confidence interval (CI): 0.52, 0.77). *R*^2^ of the linear model is 0.35. The mother's age is also significantly correlated with the number of DNMs (*P*=7.61 × 10^−6^), with the estimated rate of increase of 0.35 DNMs per year of age (95% CI: 0.21,0.51), after controlling for father's age (Table 1a). The Bootstrap 95% CI with 1,000 replications for the two regression slopes are: (0.51, 0.78) and (0.19, 0.50) for father's and mother's age, respectively. Because the father's and mother's ages are highly correlated with each other (Pearson's *r*=0.70), we performed permutation analysis to test whether the parental ages are independently correlated with the number of DNMs (Supplementary Fig. 6). The results showed that the maternal age effect on the DNM rate was not likely to be random. Furthermore, we investigated the parental age effects of DNMs at CpG sites and non-CpG sites. The maternal age effect is almost as strong at the non-CpG sites (*P*=8.39 × 10^−6^), but is not significant at the CpG sites. On the other hand, father's age is significantly correlated with number of DNMs at both CpG and non-CpG sites (*P*=1.33 × 10^−5^ for CpG sites and *P*<2.00 × 10^−16^ for non-CpG sites). This is consistent with the hypothesis that there is a lower rate in deamination in CpG sites in females than males, or that mutations are better repaired in females in CpG sites^5^. However, with only 3,894 DNMs in CpG positions in 693 families (∼5.62 per trio), assuming that the majority of the mutations came from the father, we may not have enough statistical power to observe a significant maternal effect. In the future, a larger and phased data set may provide a more definite answer. In addition, we regressed the number of DNMs in each chromosome on father's age and the difference between the parents' ages. Both parents' ages are significant at chromosome 5, 7 and 16; father's age is the only significant factor in 14 chromosomes, and neither parents' ages are significant in 5 of the 22 chromosomes (the smaller ones), at 0.05 level (Fig. 2).

To test whether the observed maternal age effect was caused by just a subset of the chromosomes, we performed multiple linear regression on the total number of DNMs by omitting chromosomes 5, 7 and 16. Both parents' age are significant in this case (*P*<2 × 10^−16^ for father's age and *P*=6.8 × 10^−4^ for mother's age), indicating that the maternal age effect is not limited to these three chromosomes only.

### Parental origin of de novo mutations

To disambiguously test for maternal age effect on germline *de novo* mutations, we used DNMs found in male newborns in the X chromosome and phased DNMs in the autosome. The germline *de novo* mutations in the X chromosome in the male probands are, in theory, of maternal origin. We generated the 153 putative DNMs in 359 male probands conceived without ART (mean=0.43, where 239 probands have no DNMs detected, Supplementary Data 1). We then fit multiple linear regression on the number of DNMs versus both parents' ages; neither parents' ages are significant at 0.05 level. Mother's age has a nominal *P* value of 0.04 when it is entered alone into the linear regression.

**Figure 3:**
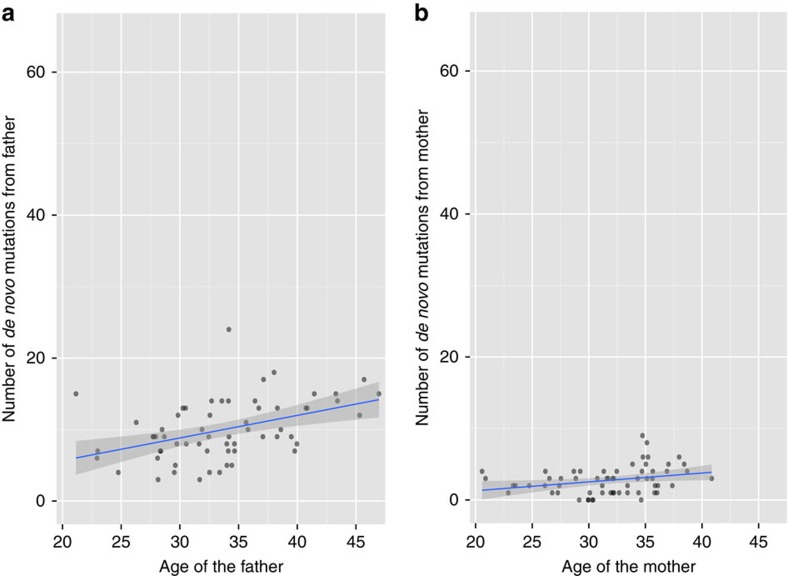
Scatter plots with linear regression line on parental ages and their respective number of *de novo* mutations in the 61 trios with Illumina sequencing data. (**a**) The number of DNMs of paternal origin is plotted against the father's age (in years). The blue line shows the linear fit (estimate of the slope=0.31, *P*=5.15 × 10^−4^) and the grey band represents the 95% confidence interval. (**b**) The number of DNMs of maternal origin is plotted against the mother's age (in years), the blue line shows the linear fit (estimate of slope=0.12, *P*=0.02), and the grey band represents the 95% confidence interval.

We determined the parental origin of autosomal DNMs detected using Illumina sequences in the 61 trios. We were able to determine the parent-of-origin in 30% of the DNMs (776 out of the 2,573 DNMs called by both Genome Analysis Toolkit (GATK) and Strelka pipelines) using our custom scripts in addition to modules PhaseByTransmission and ReadBackedPhasing in GATK. On average, the father transmits 3.44 times more DNMs than the mother does (that is, on average, 78% of the total number DNMs came from the father and 22% of those came from the mother, although the proportion of DNMs of paternal origin varies between 0.5 and 1; Supplementary Fig. 2). This ratio is consistent with previous studies, 3.9 reported by Kong *et al.* and 3.1 by Francioli *et al.* Following this, we fit simple linear regression models to the normalized number of DNMs (observed number divided by the proportion of DNMs phased for the proband) from the father and the mother with their respective ages (Fig. 3). The father's age is significantly correlated with the number of DNMs passed on from the father (estimated increase in rate per year=0.31, *P*=5.15 × 10^−4^, simple linear regression). Furthermore, our analysis indicated that the mother's age also shows a correlation with the number of DNMs passed on from the mother (estimated increase in rate per year=0.12, *P*=0.02).

To validate our pipeline, we compared the DNMs with parent-of-origin in NA12878 determined using our pipeline with Supplementary Table 1 in a study by Conrad *et al.*^6^, where 44 of the 49 germline DNMs were determined for their parent-of-origin. One of these 44 DNMs could not be lifted over from hg18 to hg19. We were able to phase 12 of these DNMs in our pipeline, of which 11/12 sites were concordant. One site was determined to have a maternal origin in the original paper^6^, but was assigned a paternal origin in our pipeline. Supplementary Figure 5 shows the DNM site with discrepancy with Conrad *et al.* We estimate that the specificity of our parent-of-origin caller based on GATK's ReadBackedPhasing is around 11/12=91.7%.

Our observation on the impact of maternal age on number of maternally derived DNMs contradicts the observations in Francioli *et al.*^3^ (*P*=0.94 between number of DNMs with maternal origin and mother's age); we suspect that these different results could be due to the difference in the age distributions in the two cohorts. The Francioli *et al.* study stated that the average age for the fathers at conception was 29.4 years, which in our cohort was 33.4 years. While the average age of the mothers was not mentioned in the former manuscript, we estimate based on the paternal age that it is on average 4 years younger than the mothers in our cohort. We will examine the differences in younger versus older parents in the next section.

### Mutation rate per year change with parents' ages

**Figure 4:**
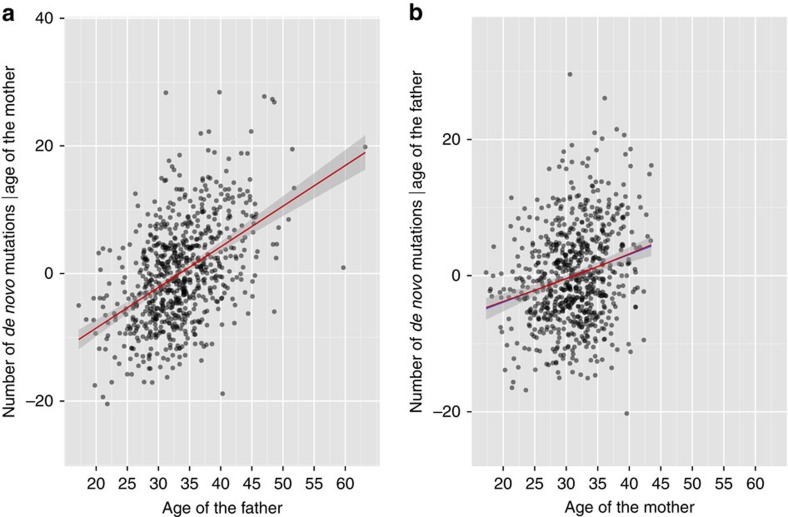
Partial residual plots on parental ages and number of *de novo* mutations. (**a**) The residuals derived from regressing the number of DNMs on the mother's age are plotted against father's age. The blue line shows the linear fit, the grey band represents the 95% confidence interval. The red line shows the best fit using the Generalized Additive model based on the Generalized Cross Validation (GCV) score. (**b**) The residuals from regressing the number of DNMs on father's age, plotted against mother's age. The estimated effective degrees of freedom for father's and mother's ages are 1.00 and 1.19, respectively.

Interestingly, while Kong *et al.* indicated that an exponential model was favoured for the paternal age effect on the number of DNMs^2^; Francioli *et al.* showed that a linear model fit the data better by estimating λ in the Box–Cox transformation^3^. We also fit an exponential model to our data (Table 1b). While we cannot compare the linear and exponential models directly, we note that the *R*^2^ for the exponential model (0.34) is slightly smaller than that of the linear model (0.35). Interestingly, the *P* value for the mother's age in the exponential model is more significant than that in the linear model. We further investigated the non-linear effects on parental ages by a Generalized Additive Model (GAM)^7^, where the number of DNMs depends on the smooth functions of both father's and mother's ages, optimized by the Generalized Cross Validation criterion. While both of the smoothing parameters for parental ages are significant (Table 2), the estimated effective degrees of freedom for the smoothing parameter for father's age is 1.00 and for the mother's age is 1.19. Indeed, the residual deviance of fitting a GAM with a linear and a smoothing term for father's age, while keeping the smoothing term for the mother's age is 0.01, indicating little difference in the two models. In contrast, the residual deviance of fitting a GAM with a smoothing term for mother's age, while keeping the smoothing term for the mother's age is 21.15 lower than fitting a linear term to mother's age, and has a higher Bayesian Information Criterion (4,706.45 vs 4,705.62), indicating a small non-linear relationship between the number of DNMs and the mother's age. The partial residual plots from MLR and GAM are shown in Fig. 4.

We next sought to identify if there is any difference in the increase in mutation rate per year in younger and older parents by splitting the data in half using either the median father's age (32.9 years) or the median mother's age (31.3 years) and fit MLRs on both parents ages on the total number of DNMs (Supplementary Table 2). Interestingly, estimates of the slope for father's age are quite similar in older (>median father's age) and younger (<median father's age) fathers (0.67 vs 0.71, 95% CI: 0.47, 0.87 vs 0.43, 0.99). On the other hand, the estimate of the slope in older mothers is almost double of that in the younger mothers (0.61 vs 0.31, 95% CI: 0.28, 0.94 vs 0.04, 0.58), after accounting for the father's age. This observation suggests that while the germline mutation rate increases linearly with father's age, it accelerates in older mothers.

### Batch effect

The genomes used in this study were sequenced by CGI between October 2011 and August 2013 with pipeline software versions 2.0.0–2.0.4. Different experimental procedures and software versions may affect the number of bases being mapped to the reference genome (‘Gross mapping yield') and fraction of bases that are called with high confidence (‘Fully called genome fraction VQHIGH') and, in turn, change the effective number of bases being called for each trio. That is, the more called bases with high confidence may lead to higher number of DNMs called and *vice versa*. We, therefore, investigated whether there is significant batch effect in the *de novo* mutations calling by linear regression. Indeed, in the unfiltered data, software version 2.0.3, and fully called genome fraction are both significant (*P*=2.34 × 10^−5^ for software version 2.0.3 and *P*=0.03 for ‘fullyCalledFractionHIGH', Supplementary Tables 3 and 4). The ‘GenomeCoverageGrossMappingYield' is not significant (*P*=0.17 and *P*=0.47) before or after filtering (Supplementary Table 5). Furthermore, we see that the estimated number of DNMs is higher in software version 2.0.3 in the unfiltered data (Supplementary Fig. 3a). On the other hand, such effects are not apparent with the filtered data (Supplementary Fig. 3b), indicating that our filtering steps may satisfactorily remove the batch effect. To completely eliminate the possibility that Software versions 2.0.0 and 2.0.3 are responsible for the maternal age effect we are observing, we fit the MLR model excluding software versions 2.0.0 and 2.0.3, and found that both parents' ages are still significant (*P*<2.00 × 10^−16^ for father's age and *P*=1.97 × 10^−5^ for mother's age).

### Validation of the *de novo* mutations

To validate the DNMs we detected with the CGI platform, we performed three separate analyses. First, 61 of the 693 trios underwent WGS at ∼40X through Illumina Services. Second, one additional family (parents and their monozygotic newborn twins) underwent sequencing by both Illumina and CGI platforms. Third, Sanger sequencing was performed on a selected set of variants detected by either the CGI or Illumina Platform.

We estimated the sensitivity and specificity of our DNM analysis pipeline using the 61 trios in our cohort that were also sequenced by Illumina. That is, we validated close to 9% of our data using an orthogonal and reliable technology. We developed two analysis pipelines for discovering DNMs using GATK^8^ and Strelka^9^, respectively, with the Illumina sequences and alignments.

**Figure 5:**
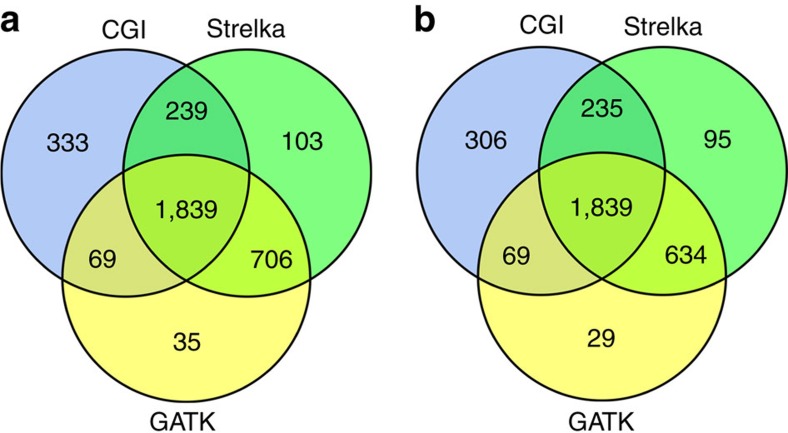
Comparison of the three DNM discovery pipelines with Venn diagrams. (**a**) The overlap between the list of DNMs called in the 61 trios by CGI custom pipeline (blue circle), Strelka custom pipeline (green circle) and GATK PhaseByTransmission custom pipeline (yellow circle). 1,839 DNMs were called in all 3 custom pipelines and 2,583 DNMs were called in at least 2 custom pipelines. There are 333 (13% of total called by CGI) DNMs uniquely called by the CGI custom pipeline, 103 DNMs (4%) uniquely called by the Strelka custom pipeline and 35 DNMs (1%) uniquely called by the GATK PhaseByTransmission custom pipeline. (**b**) The overlap between the list of DNMs called in the 61 trios in sites that are considered callable by all 3 custom pipelines, with the same colour scheme as in **a**. There are 306 (3% of total called by CGI) DNMs uniquely called by the CGI custom pipeline in commonly called bases, 95 DNMs (5%) uniquely called by the Strelka custom pipeline and 29 DNMs (1%) uniquely called by the GATK PhaseByTransmission custom pipeline.

Using variant calls from our custom pipelines with Strelka and GATK from Illumina data, as well as the variant calls from the custom CGI analysis pipeline, we define our truth set to be those DNMs that were called by at least two custom pipelines (Fig. 5). We then estimated the sensitivity for our CGI custom pipeline to be 75% and the specificity to be 87%.

The resulting list contains 2,918 DNMs from Strelka and 2,679 from GATK PhaseByTransmission from the 61 trios. The comparison between the three pipelines is summarized in the Venn diagram (Fig. 5a). However, since CGI and Illumina data have their own characteristics in terms of context-dependent error rate, GC bias and genome coverage, the total number of DNMs detected may not truly reflect the accuracy of each of the custom pipelines. We, therefore, created the lists of commonly called bases for each trio on each platform and compared the sensitivity and specificity of each of the pipelines in each trio only in the regions of bases called by all three custom pipelines in all three members of the trio (Fig. 5b).

For the family with the monozygotic twins, we summarize the results in Supplementary Fig. 4. Since they are monozygotic, we hypothesize that they share all the germline *de novo* mutations. Supplementary Figure 4b,c and 4d show the concordance between the two twins in the custom CGI, Strelka and GATK pipeline, respectively. Supplementary Figure 4a shows the comparison of the shared variants called within each pipeline. 93% (43 out of 46) of the DNMs found in both twins are also detected by one of the Illumina pipelines. While this is only one family, it further confirms that our CGI pipeline is accurate.

Furthermore, we validated two set of variants using Sanger sequencing. The first set contains a randomly selected set detected with the CGI custom pipeline only (which were considered as false positives in estimation of specificity of the CGI pipeline), 39 of these sites were successfully run on all members of the trios. Of these 39 sites, 27 of the 39 (69%) are true positives, which indicates that we have underestimated our specificity. The second set contains the DNM sites that were called by both the Strelka and GATK pipelines only (not in CGI) in the monozygotic twins. Of the 12 that we were able to successfully sequence, 11 sites were validated, while 1 variant was not confirmed in the proband (false positive). This suggests that our measure of sensitivity (by assuming sites that are called by both Strelka and GATK pipelines are true positives) is largely correct (Supplementary Table 6).

### Mutation rate estimation

We estimated the mutation rate by accounting for the sensitivity and specificity of the CGI pipeline and the effective number of base pairs in the study. We estimated there were on average 1.05 × 10^−8^ mutations per nucleotide per generation, well within the range of mutation rates described in previous studies^2^,^6^,^10^,^11^. Furthermore, as previously shown, the transition rate at CpG sites is more than 10 fold more frequent than that in the non-CpG sites (Supplementary Table 7). We estimated the true slope for father's age on number of DNMs in autosomes to be 0.92 DNMs per year (95% CI: 0.74, 1.10) and that for the mother's age to be 0.51 DNMs per year (95% CI: 0.29, 0.73). The true slope for father's age for the phased DNMs is estimated to be 1.67 DNMs per year (95% CI: 0.76, 2.59) and that for the mother's age to be 0.66 DNMs per year (95% CI: 0.09, 1.22). The variability on the proportion of DNMs phased in each family has increased the s.e. of our estimates substantially.

We would like to emphasize that while we attempted to account for all possible sources of biases when estimating the mutation rates, and while our estimated numbers concordant with previous studies using next-generation sequencing technology, there remain many uncertainties in the reference genome as well as in the sequencing technologies that cause challenges in yielding precise and highly accurate numbers.

## Discussion

Although the correlation we found between the mother's age and the number of DNMs is statistically significant, there are several potential technical and biological explanations that could have led to a falsely significant result. We showed that if not handled carefully, sequencing batch effect can either cause spurious correlation or reduce the power of detecting real ones. As the sample size of the genomic studies grows and often cross institutions, checking and minimizing batch effects may be one of the most important quality control steps before any further analysis.

Mosaicism in one or both parents could also lead to false correlation. If the mosaic parent has a large deletion, or underwent a copy neutral loss of heterozygosity event in most of the cells, it would appear that the parent is homozygous in the region. As a consequence, germline single-nucleotide variants (SNVs) in the newborn inherited from that parent would appear as DNMs. Since the prevalence of mosaicism is known to be correlated with age^12^, this type of error would introduce an apparent positive correlation between parental ages and the number of DNMs passed on to the child. To test whether mosaicism occurred in our cohort, we removed clusters of nearby putative DNMs in the same newborn from 10 bp, 10^2^ bp, ..., up to 10^8^ bp, and analysed the linear fit of the number of DNMs versus father's age and mother's age for each set. We found that both parents' ages remained significantly correlated with total number of filtered DNMs even when we removed SNPs that are within 1 million bp from each other (*P*<0.05, Supplementary Table 8). Neither parent's age is significant when the cluster size is 100 million bp or larger, which is longer than 7 of the autosomes in the human genome. Furthermore, from our orthogonal validation with Sanger sequencing, we realized that most of our false-positive DNMs are due to the variant not being present in the proband, instead of the variant being present in one of the parents (Supplementary Table 6). Another possible source of a falsely significant result is the possibility that advanced maternal age increases the rate of postzygotic somatic mutations in the offspring. Therefore, there would be decreased genomic integrity in the offsprings of older mothers. Somatic mutations that happen after the multi-cell embryo stage would often exhibit smaller allele frequencies in the blood (frequencies <<0.5). We, therefore, filtered out variants that significantly violated the heterozygous diploid assumption in the analysis pipeline. However, we note that in our data set it is not possible to distinguish germline DNMs in the parents from very early somatic mutations in the newborns (that is, when the somatic mutation allele has a similar allele frequency to the reference allele). False negatives could also introduce the apparent relation between maternal age and number of DNMs if the false-negative rate is correlated with maternal age, as most false negatives were likely introduced by excluding sites with a large proportion of no-calls (>1% in the cohort) or by eliminating variants in the probands that have no-calls in a parent. However, this would be expected to yield a negative correlation of the number of DNMs with both parents' ages, which is not observed in our data. We also investigated the effect of various filtering steps on the association with maternal age effect (Supplementary Tables 1, 8, 9 and 10). The results indicate that the maternal age effect is indeed robust with the filtering we applied.

In this study, we confirmed in a racially and ethnically diverse population that the DNM rate is dominated by the father's age; we also showed evidence that the mother's age plays a small but significant role. Previous studies of WGS data did not find a statistically significant correlation with maternal age. This discrepancy is most likely due to the lack of sufficient statistical power from a smaller sample size, as well as a greater number of older mothers at conception in our cohort. However, Kong *et al.* did show that there was some over-dispersion in the number of maternal *DMNs* in the five families in which they were able to phase the proband's genome^2^. While human maternal age has previously only been correlated with large chromosomal anomalies, previous studies in the mouse have used microsatellite data to show an increase in cell divisions in the oocyte with increasing age^13^. We also showed that the rate of increase in germline mutations accelerates with maternal age while being constant with paternal age. The increase in the number of DNMs in the proband with increasing paternal age is thought to result from an accumulation of mutations with increasing number of cell replications. The germ cells of women are thought to be relatively quiescent, though a possibility of maternal age effect has been suggested^14^. The results presented here suggest three possibilities: the female germ cells are more prone to accumulate spontaneous mutations over time than currently thought; or that the zygotes from older mothers' germ cells have higher mutation rate; or oocytes from older mothers undergo more cell divisions. These observations provide greater insights in our understanding of reproductive biology.

## Methods

### Data

The 774 complete family trios and quads, where applicable from twin births, (father, mother and newborn(s)) with validated pedigrees are from an Institutional Review Board-approved WGS-based study on the causes of preterm birth (Vockley *et al.*, 2015, Manuscript in preparation). Informed consent was obtained for all subjects in this study, which includes cases (preterm births) and controls (fullterm births). Sample batches were sent to CGI (Mountain View, CA) for WGS (see Bodian *et al.*, 2014 on sample collection^15^). Complete Genomics' Assembly (CGA) Pipeline versions 2.0.0–2.0.4 with the NCBI build 37 human genome reference^16^ were used for genome assembly and variant calling. The small variants (SNVs and Insertions/Deletions) were merged into a single VCF file using mkvcf beta version from CGA tools version 1.6.0 (refs 15, 17).

Of the 774 families in the preterm birth study, we excluded 55 families having multiple births, 25 families with conception involving ART, and 1 outlier by Bonferroni Outlier Test (Supplementary Fig. 1). Six hundred and ninety-three families with singleton births were used in this study, of which 208 of the newborns were born preterm (gestational age <37 weeks).

Sixty-one of the trios and a quartet were sent to Illumina Inc., (San Diego, CA) for WGS and assembly (pipeline version 2.0.3).

### Generating the list of *de novo* mutations in CGI data

The list of putative DNMs in the autosomes from the 719 singleton births (including those that were conceived with natural and assisted methods and the outlier) were generated as follows:

The initial putative DNMs list consists of SNV sites in the autosomes that are biallelic across the cohort, with call rate ≥0.99, and where the variant is observed in one of the probands only in the entire cohort (2,434 individuals), with variant classified as GQHIGH at the proband, CGI refScore >0 in both parents and with variant read count frequency ≥0.3;

To remove potential false positives caused by mapping error, those putative DNM sites with alignability (36 mers, Guigo—CGR, Barcelona, obtained from UCSC Table Browser) <1 are removed^18^;

Putative DNMs within 1,000 bp in the same proband are removed because they might be caused by miscalling or a different mutation mechanism (for example, somatic hemizygous deletion in one of the parents);

To remove those false-positive sites that are more likely to be common polymorphisms in the population, variants that are present in dbSNP 131 (ref. 19), the Exome Sequencing Project^20^ or the 1000 Genomes Project Phase 1 (ref. 21) are removed;

Variants that are annotated by CGA tools as in Segmental Duplication are removed;

Putative variant sites are annotated using Gemini^22^ and custom scripts. Those sites that are in a RepeatMasker^23^ tandem repeats region predicted by Tandem repeats finder^24^ are removed to avoid mapping errors;

Finally, those sites with less than five reads covering either allele or >5% reads representing a third allele are removed.

The list of putative DNMs in chromosome X from the 368 male singleton births (of which 9 were conceived with ART) were generated similarly to the autosomes, except for the last step, where we require the total number of reads covering the site to be at least five and the read count frequency for the reference allele <5%.

### Generating the list of *de novo* mutations in Illumina data

To obtain a list of DNMs from the Illumina WGS data, we developed two independent customized pipelines using Strelka^9^ and GATK^7^ (HaplotypeCaller and PhaseByTransmission).

Strelka was originally developed to detect small somatic variants in tumour samples by jointly modelling the tumour samples with the normal samples. One of the advantages of Strelka is that it performs local realignments on both tumour and normal reads at the same time, greatly reducing the number of false positives caused by misalignments. Most of the metrics associated with each variant are based on either tier 1 or tier 2 filtering. While tier 1 is more stringent, tier 2 allows users to detect low-quality reads that show discrepancies from the assumptions. We obtained the list of putative DNMs in each proband in the Illumina data using Strelka Version 1.0.14 by subtracting each proband from each of the parents separately (that is, treating the proband as the ‘tumour' and the parents as the ‘normal' genome), and taking the intersection of the two sets. We then further filter the variants by requiring that:

All members of the trio have at least 10 reads covering the site;

The number of basecalls filtered is <20% of the original read depth for tier 1;

There is ≤1 read with deletions spanning this site at tier 1;

For either parent, the number of reference reads is ≥90% of the total number of reads covering the site; with no reads with the putative DNM found in the parents. Also, the number of reads with reference bases at tier 1 is at least 80% of that at tier 2;

For the proband, the number of reads with the genotype called is at least 90% of the total number of reads covering the site. Also, the number of reads with reference bases at tier 1 is ≥80% of that in tier 2; the same applies to the alternative bases;

For the proband, the number of reads with the minor allele is ≥30% of the total number of reads covering the site;

Sites within 1,000 bp in the same proband are removed;

Those putative DNM sites with alignability (36mers, Guigo—CGR) <1 or in a RepeatMasker tandem repeats region predicted by Tandem repeats finder are removed^23^;

Finally, to remove those false-positive sites that are more likely to be common polymorphisms in the population, variants found in dbSNP 131 (ref. 19), the Exome Sequencing Project^20^ or the 1000 Genomes Project^21^ are removed;

We ran the HaployperCaller module in GATK Version 3.2.2 on each family followed by the PhaseByTransmission module in GATK with default setting. The HaplotypeCaller module performs local realignments on each family member, hence reducing the false positive rate introduced by misalignment, and ensures that calls are consistent within each family. The PhaseByTransmission module phases the variant calls by familial transmission as well as identifies any calls that violate Mendelian inheritance. We then filter the variants in the Mendelian Violations File from the PhaseByTransmission module by requiring that:

TP (Transmission Probability) has to be greater or equal to 30;

All members of the trio have at least 10 and at most 100 reads covering the site;

The PL (likelihood of the genotype) of the homozygous reference genotype for both parents should be equal to 0; and the PL of the heterozygous genotype for the proband should be equal to 0;

For the parents, the number of reads with the alternative allele should be equal to 0;

For the proband, the number of reads with the reference allele and with the alternative allele have to be greater than or equal to 5, respectively;

For the proband, the number of reads with the minor allele is ≥30% of the total number of reads covering the site;

Sites within 1,000 bp in the same proband are removed;

Those putative DNM sites with alignability (36 mers, Guigo—CGR) <1 or in a RepeatMasker tandem repeats region predicted by Tandem repeats finder are removed^23^;

Finally, to remove those false-positive sites that are more likely to be common polymorphisms in the population, variants found in dbSNP 131 (ref. 19), the Exome Sequencing Project^20^ or the 1000 Genomes Project^21^ are removed.

### Determination of parental origin of the DNMs

Parental origin of a DNM can be determined if a haplotype block from haplotype assembly (haplotype phase from sequencing data) covers both the DNM and a phase informative site. Haplotype blocks 20 kb up- and downstream of the true DNM sites were generated with the ReadBackedPhasing module in GATK, using the BAM files mapped by Illumina Services and the variant calls from the PhaseByTransmission module in GATK. A DNM is considered of maternal origin if the DNM allele is on the same phase as a maternally inherited haplotype determined by transmission, and *vice versa*. If there is conflict between the haplotype phase from analysing the sequencing data versus analysing the familial transmission information, or if the haplotype block containing the DNM does not include a phase informative site, the parent-of-origin of the DNM cannot be determined. Overall, we were able to determine the parent-of-origin of 31% of the putative DNMs detected by both GATK and Strelka (963 out of 3,128 sites). We confirmed the accuracy of our parent-of-origin pipeline by running it on the NA12878 sample from Illumina Platinum Genomes^25^ (Supplementary Fig. 5).

To validate our *de novo* mutations pipeline for genomes sequenced by Illumina, we tested it on the NA12878 trio (NA12878 and parents NA12891 and NA12892) in the CEPH pedigree 1463, which are part of the Illumina Platinum Genomes project^26^. The aligned BAM files are downloaded from European Nucleotide Archive with accession number ERP001960. We ran the same GATK phasing pipeline as described in the Methods section and generated 2,139 *de novo* single-nucleotide mutations and were able to phase 686 of them (33%), of which 363 are from the father (53%) and 323 are from the mother (47%). Note that this list includes somatic mutations in the cell line of NA12878, which is not possible to distinguish from germline mutations without extra information such as whether the variant is inherited in her 11 offsprings. Thus, we do not see a biased proportion of variants of paternal origin. Tweleve of these phased DNMs were confirmed as germline DNMs with determined parent-of-origin^6^. We, therefore, compare our findings with the results in Conrad *et al*.

### Statistical methods for parental age effect

All statistical analysis were performed using R 3.0.2 (‘Frisbee Sailing'). We use a linear model to test the parental age effects and adjust for natural conception (versus conceived using ART) and preterm status. The model used is:


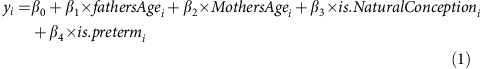


where *y*_*i*_ is the number of DNMs observed in *i*th proband, *β*_0_ denotes the intercept, fathersAge_*i*_ is the age of the father of the *i*th proband, mothersAge_*i*_ is the age of the mother of the *i*th proband, is.NaturalConception_*i*_ is an indicator variable on whether the *i*th proband was conceived naturally, and is.preterm_*i*_ is an indicator variable on whether the *i*th proband was born preterm. *β*_*j*_,*j*=1–5, are coefficients of those variables.

After removing those probands that are conceived with ART, we refit the model as follows:


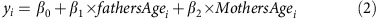


The variables and coefficients are defined the same as above.

The exponential model was fitted as follows:


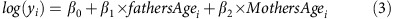


The variables and coefficients are defined the same as above.

The linear model for per chromosome analysis was fitted as follows:


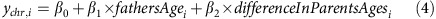


where y_chr*,i*_is the number of DNMs observed in chromosome chr in *i*th proband and differenceInParentsAges_*i*_=mothers Age_*i*_−fathers Age_*i*_.

### Batch effect

We obtained ‘SOFTWARE VERSION', ‘Fully called Genome Fraction VQHIGH' and ‘Gross mapping yield (Gb)' from the summary files provided by CGI for each genome. We then look for the effect of ‘SOFTWARE VERSION', ‘Fully called Genome Fraction VQHIGH' and ‘Gross mapping yield (Gb)' by including them in the linear models of number of DNMs versus parental ages. The models are specified as follows:


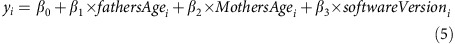



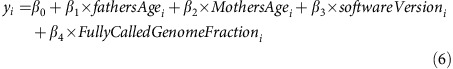



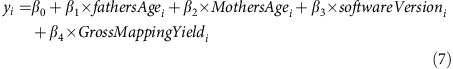


### Maternal age

Because the father's and mother's ages are highly correlated with each other (Pearson's *r*=0.71), we calculated the variance inflation factor. The variance inflation factor for both father's and mother's ages is 1.99 (<5), indicating that the multicollinearity issue is minimal for this ordinary least squares regression analysis.

To further test whether the correlation of mother's age with the number of DNMs in the proband is due to chance, a permutation test was performed. If mother's age has no effect on the total number of DNMs, we should be able to shuffle mother's ages within the data set, while keeping the correlation between the paternal and maternal ages high, and obtain the same significance for the maternal age effect. We, therefore, generated 10,000 sets of simulated maternal ages by randomly shuffling the differences in age between the father and mother in each family, and calculated the new *t* value for maternal age in each set (Supplementary Fig. 6). None of the randomized set has a *t* value as high as that in the original data set (4.58); that is, the chance of observing this value or higher is <0.0001, indicating that it is highly unlikely that the maternal age effect we observed is due to chance.

### Mutation rate estimation

For CGI data, we randomly selected 500 individuals from our cohort and generated a list of sites that have no-calls in at least 1% of the individuals. We used subtractBed in BEDTools^27^ to obtain the commonly callable region from the autosomes in UCSC hg19, estimated to be 2.21 billion base pairs. We also calculated the average number of bases with no-calls in these individuals (91.18 million base pairs). On average 1/4 of the no-call sites in an individual are shared in over 90% of the same selected individuals (22.99 million base pairs), indicating that there is high degree of variation in the sequencing coverage along the genome in the individuals. The estimated number of commonly callable base pairs is an upper bound due to the variability in coverage between individuals. During our filtering step we also remove those sites that are in the predicted tandem repeats regions, those that are in segmental duplication regions, and those with alignability smaller than 1; we subtract those sites from the commonly callable regions using subtractBed to generate the effective bases called. We estimate that the effective number of bases in the autosomes to be 1.62 × 10^9^. For the 61 families sequenced by both technologies, we obtained the callable bases per trio based in CGI data by intersecting the callable bases in all three family members, again using BEDTools. For Illumina data, we used the ‘get_called_regions' module in gvcftools^28^ to obtain the callable region for each genome used in this analysis. The callable base pairs per trio are obtained in the same way using the callable regions in the CGI data. Furthermore, we obtained the genome positions of callable bases in the three custom pipelines in both technologies by intersecting the callable bases in CGI and Illumina for each trio; these positions are used as the common callable bases when estimating the sensitivity and specificity of the CGI pipeline, and for Fig. 5b.

The mutation rate per nucleotide per generation is calculated as:


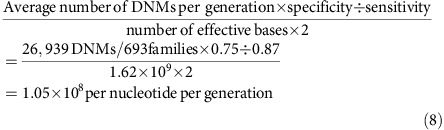


Similarly, the estimated increase in the number of DNMs per year of parents' age is calculated by adjusting for sensitivity and specificity, and normalized by proportion of effective bases called in the genome:


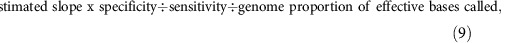


where genome proportion of effective bases called is defined as number of effective bases divided by number of bases in the reference genome hg19 without assembly gaps.

Finally, the estimated increase in the number of DNMs per year of parents' age in the phased data is calculated similarly to Equation (9), except that we further normalize the slopes by proportion of the DNMs that were phased.

### Genomic context and mutational rates

Single-nucleotide substitution rate is known to vary for different types (transition versus transversion) and in different genomic contexts (CpG sites versus non-CpG sites). In our set of 26,939 DNMs, the transition mutation rate per base per generation is 13.1 times higher at CpG sites than at non-CpG sites, and the transversion mutation rate is 2.4 times higher in this study (Supplementary Table 7). The overall rate of mutation going from a strong (G:C) base pair to a weak (A:T) base pair is 1.69 times higher than going in the opposite direction. This ratio drops slightly to 1.44 if only non-CpG sites are considered, indicating that the general trend is still true for the non-CpG sites. These observations are largely consistent with previous reported studies^2^,^25^.

The list of CpG sites was generated using a custom script on the autosomes in UCSC hg19. The effective numbers of base pairs for CpG and non-CpG positions are calculated by intersecting CpG and non-CpG positions by the callable bases in the CGI pipeline, which gives 29.04 million and 1.59 billion bp for CpG and non-CpG sites, respectively.

## Additional information

**Accession codes**: The *de novo mutation* sequencing data has been deposited at dbGap under the accession codes phs001055.v1.p1.

**How to cite this article**: Wong, W. S. W. *et al.* New observations on maternal age effect on germline de novo mutations. *Nat. Commun.* 7:10486 doi: 10.1038/ncomms10486 (2016).

## Supplementary Material

Supplementary Information Supplementary Figures 1-6, Supplementary Tables 1-10 and Supplementary Reference

Supplementary Data 1 1-based genomic coordinates for the list of de novo mutations analyzed in this manuscript. The coordinates are based on the hg19 assembly.

## Figures and Tables

**Table 1:** Regressions on the effect of parental ages on the number of DNMs.

**(a) Multiple linear regression of the total number of DNMs on the father's and mother's ages (*****R***^2^**=0.35)**					
**Model**	***β***	**s.e.**	***t***	**Pr(>*****t)***	**VIF**
(constant)	6.61	1.79	3.69	2.39 × 10^−4^	
Father's age	0.64	0.06	10.03	<2.00 × 10^−16^	1.99
Mother's age	0.35	0.08	4.51	7.61 × 10^−6^	1.99

**(b) Multiple linear regression of the natural log of number of DNMs on father's and mother's ages (*****R***^2^**=0.34)**				
**Model**	***β***	**s.e.**	***t***	**Pr(>*****t)***
(constant)	2.79	0.05	59.46	<2.00 × 10^−16^
Father's age	0.02	1.67 × 10^−3^	9.88	<2.00 × 10^−16^
Mother's age	9.3 × 10^−3^	2.04 × 10^−3^	4.58	5.64 × 10^−6^

**(c) Multiple linear regression of the number of DNMs in CpG sites on father's and mother's ages (*****R***^2^**=0.08)**					
**Model**	***β***	**s.e.**	***t***	**Pr(>*****t)***	**VIF**
(constant)	1.44	0.59	2.45	1.47 × 10^−2^	
Father's age	0.09	0.02	4.39	1.33 × 10^−5^	1.99
Mother's age	0.04	0.03	1.39	0.16	1.99

**(d) Multiple linear regression of the number of DNMs in non-CpG sites on father's and mother's ages (*****R***^2^**=0.33)**					
**Model**	***β***	**s.e.**	***t***	**Pr(>*****t)***	**VIF**
(constant)	5.17	1.61	3.20	1.44 × 10^−3^	
Father's age	0.55	0.06	9.51	<2.00 × 10^−16^	1.99
Mother's age	0.32	0.07	4.49	8.39 × 10^−6^	1.99

**(e) Linear regression model of mother's age on the number of DNMs in chromosome X in male probands (*****R***^2^**=0.01)**				
**Model**	***β***	**s.e.**	***t***	**Pr(>*****t)***
(constant)	−0.01	0.22	−0.06	0.95
Mother's age	0.01	6.95 × 10^−3^	2.03	0.04

DNM, *de novo* point mutation; VIF, variance inflation factor.

693 trios were used in the analysis in (a)-(d); 359 mother and son pairs were used in the analysis in (e).

**Table 2:** Approximate hypothesis tests on the smoothing parameters estimates in the generalized additive model of the effect of parental ages on the number of DNMs.

	**Effective degrees of freedom**	***F*** **value**	**Pr (>*****F)***
father's age	1.00	100.18	<2.00 × 10^−16^
mother's age	1.19	14.28	3.02 × 10^−5^

DNM, *de novo* point mutation.

693 trios were used in the analysis. The smoothing parameter estimates for both *P* values for father's and mother's ages are significant. The effective degrees of freedom of the estimated smoothing parameter on the father's age is 1.00 and the estimate is 1.19 for the mother's age, indicating non-linearity in the relationship between maternal age and number of DNMs.
