# Supplementary material for: New observations on maternal age effect on germline de novo mutations
Source: Nat Commun. 2016 Jan 19;7:10486. doi: 10.1038/ncomms10486 (PMC4735694; doi:10.1038/ncomms10486)
Supplement: Supplementary Information — Supplementary Figures 1-6, Supplementary Tables 1-10 and Supplementary Reference [file ncomms10486-s1.pdf]

Supplementary Figure 1. Summary of the participants used in the study.

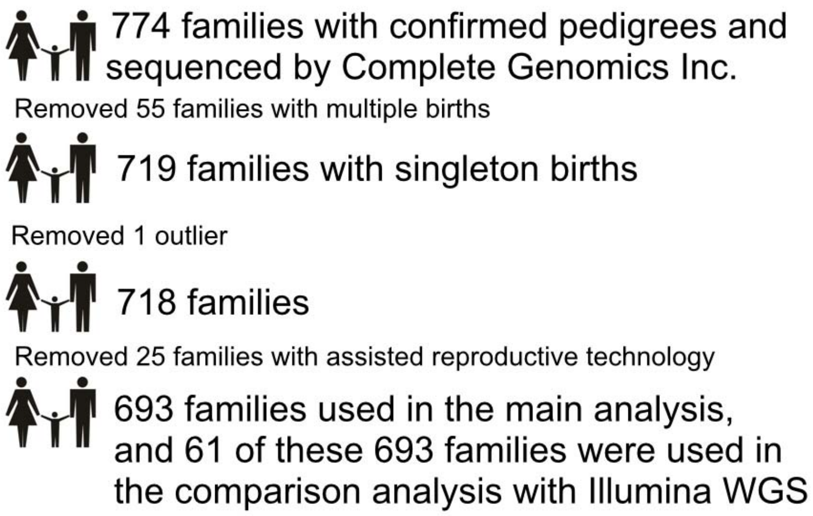

This figure shows the number of families that were included and excluded in this study.

Supplementary Figure 2. DNMs with determined parental origin in the 61 proband with Illumina Sequencing.

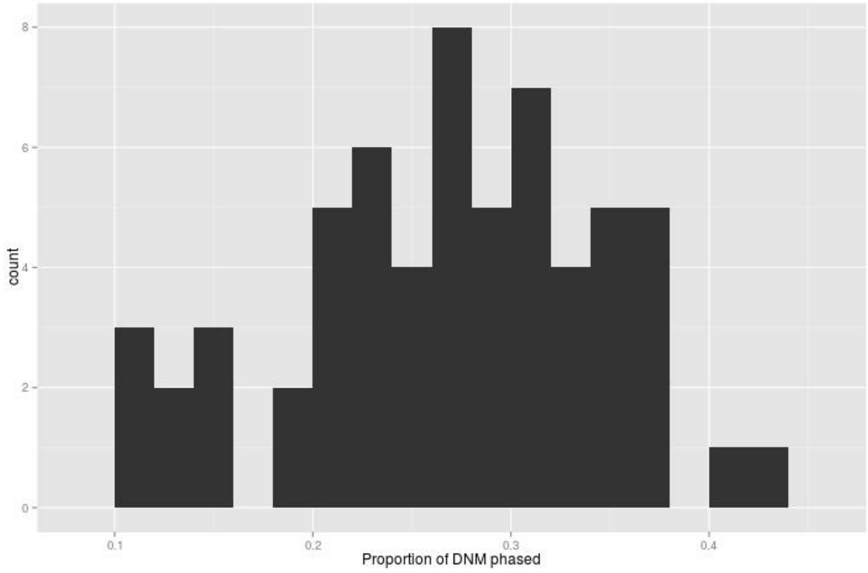

(a) the distribution of the proportions of DNMs that their parent-of-origin can be determined. On average, 27% of the putative DNMs were assigned a parental origin (range = (11% ,42%), s.d.=7.59%).

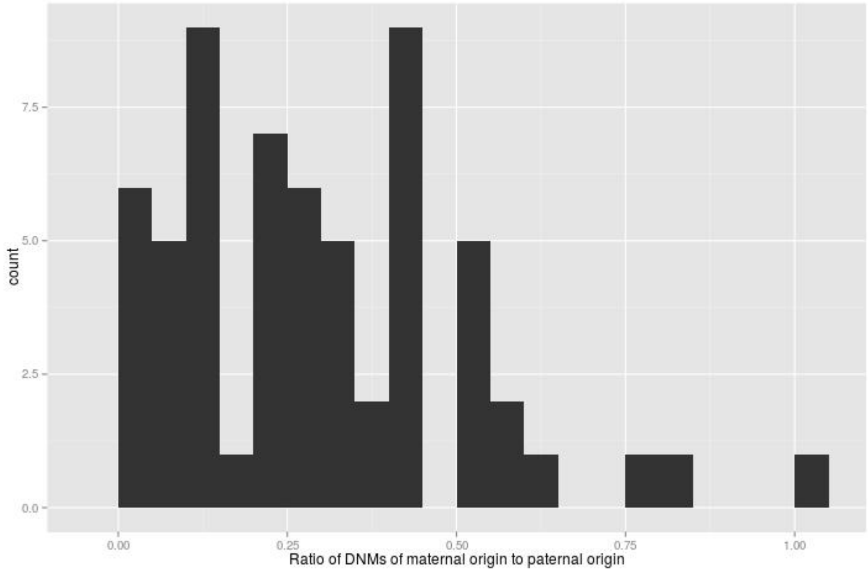

(b) the distribution of the ratios between number of DNMs of maternal origin and paternal origin. On average, the ratio between DNMs with a maternal origin and with a paternal origin is 0.29 (range=(0.00,1.00), s.d.=0.21).

Supplementary Figure 3. Number of DNMs for each software pipeline version before and after filtering.

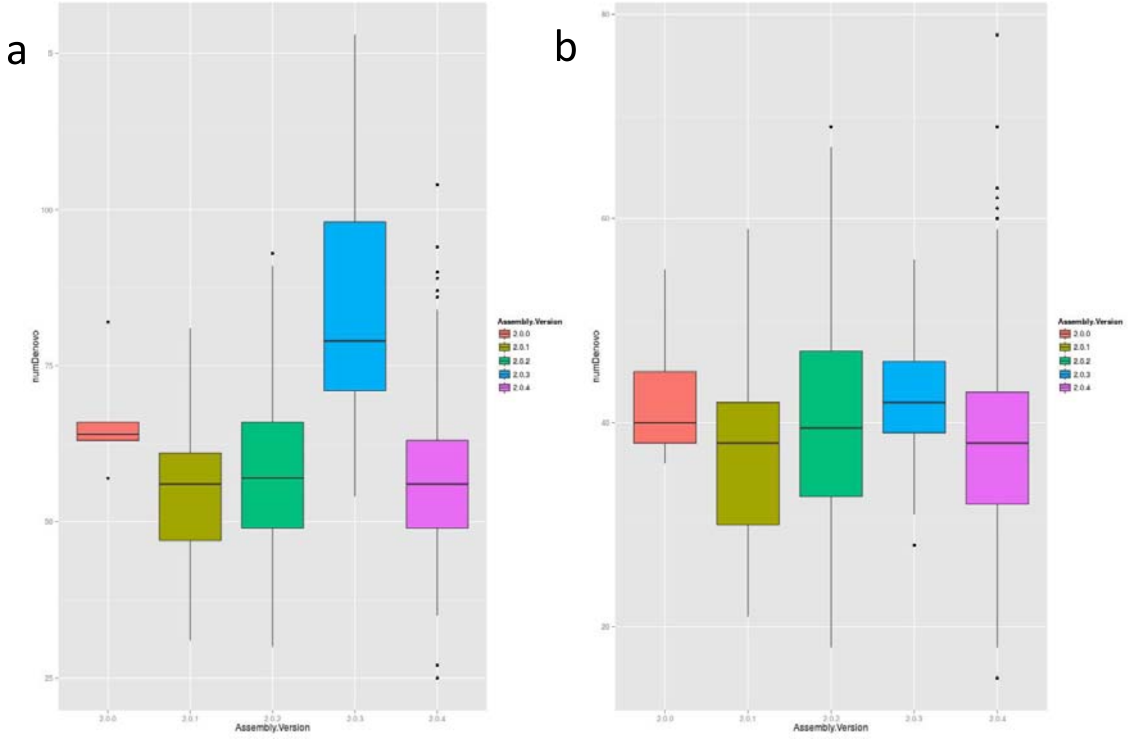

(a) shows the unfiltered data (with the outlier sample removed), where software versions 2.0.0 and 2.0.3 are showing unusually high number of DNMs. **(b)** shows the filtered data, where such trend is not apparent.

Supplementary Figure 4. Venn diagrams of the DNM sites called by CGI, Strelka and GATK custom pipeline on a family of monozygotic twins.

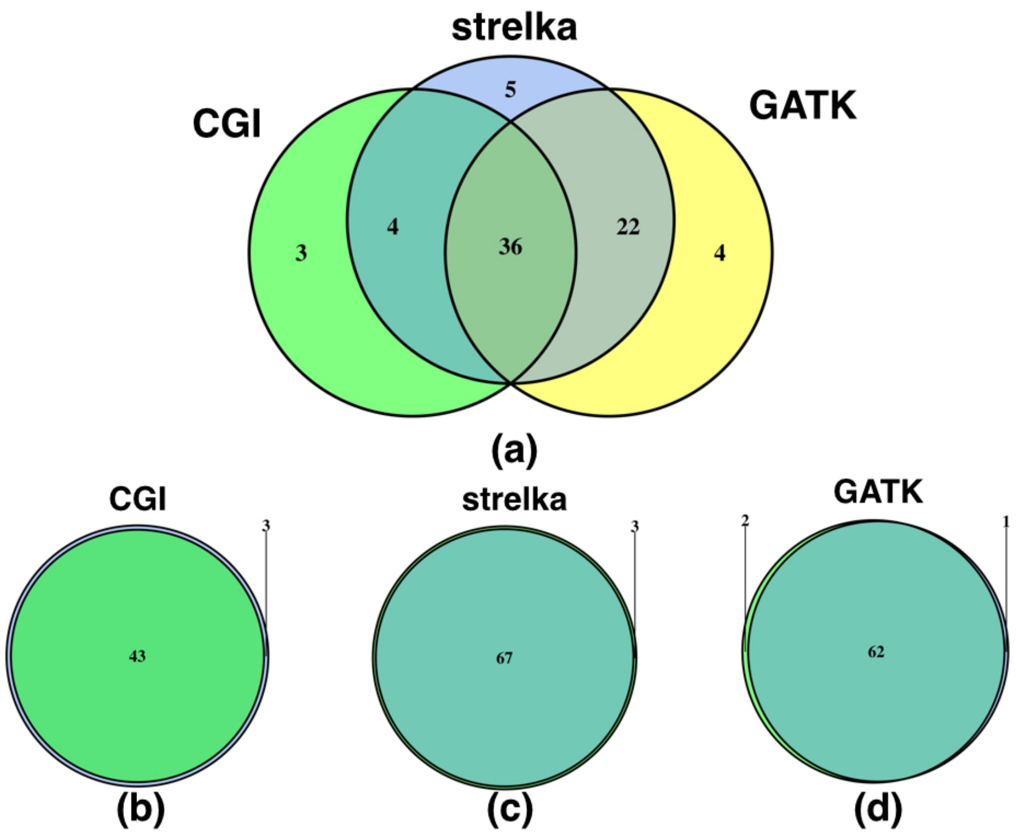

(a) shows the overlap between the 3 pipelines on the shared variants of the two monozygotic twins. **(b)** shows the overlap between the DNM calls of the two monozygotic twins from the CGI custom pipeline. **(c)** shows the overlap between the DNM calls of the two monozygotic twins from the Strelka custom pipeline. **(d)** shows the overlap between the DNM calls of the two monozygotic twins from the GATK custom pipeline.

Supplementary Figure 5. IGV screen shot of wrongly phased site chr5:52638226 in NA12878.

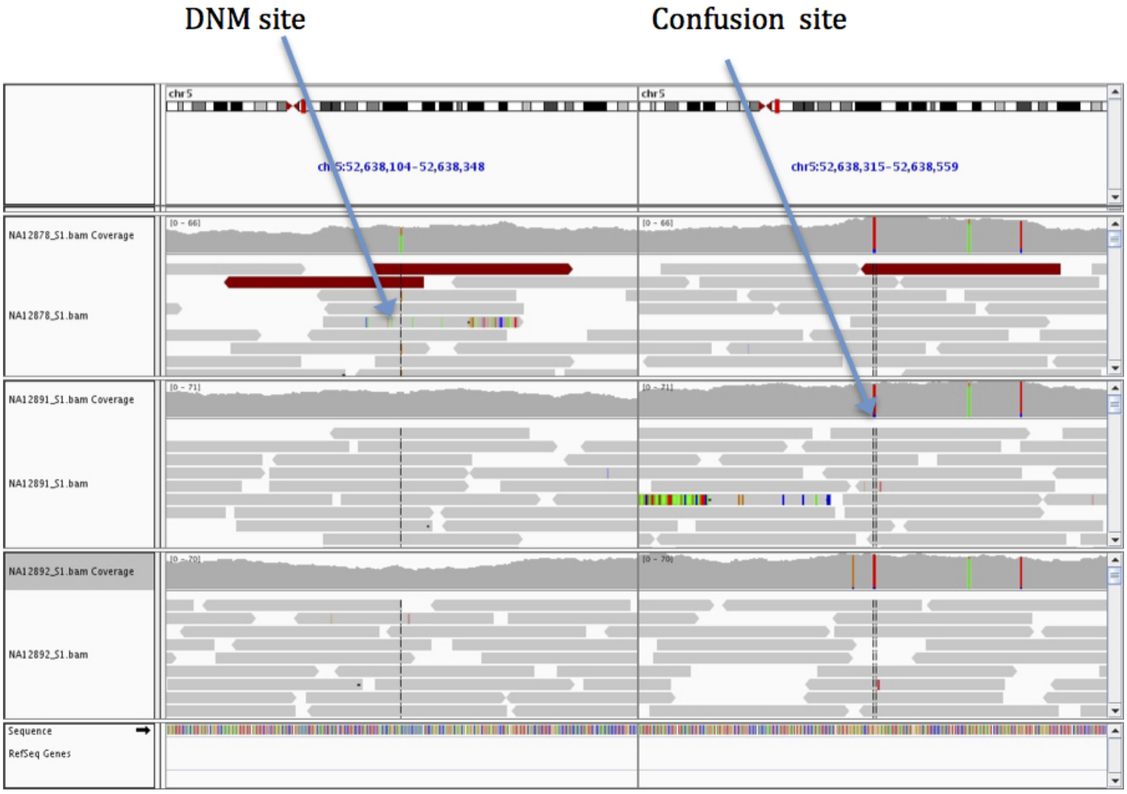

One of the 12 DNM sites from NA12878 that we were able to phase has a different parent of origin from Conrad et. *al.*, 2011[1]. This is possibly a falsely phased site from our analysis. The seemly phase informative site (confusion site in the figure) that is linked by the read pair in read is actually not phase informative, as all three members in the trio have the alternative allele. However, the father NA12891 was called by HaplotypeCaller as heterozygous whereas the mother NA12892 was called as homozygous reference due to lower percentage of reads with the alternative allele. It is worth noting that the algorithm would not make a call for parental origin if there is conflicting evidence. However in this scenario the next two heterozygous sites were deemed uninformative so a parent of origin call was mistakenly made.

Supplementary Figure 6. T values for permuted mother's age.

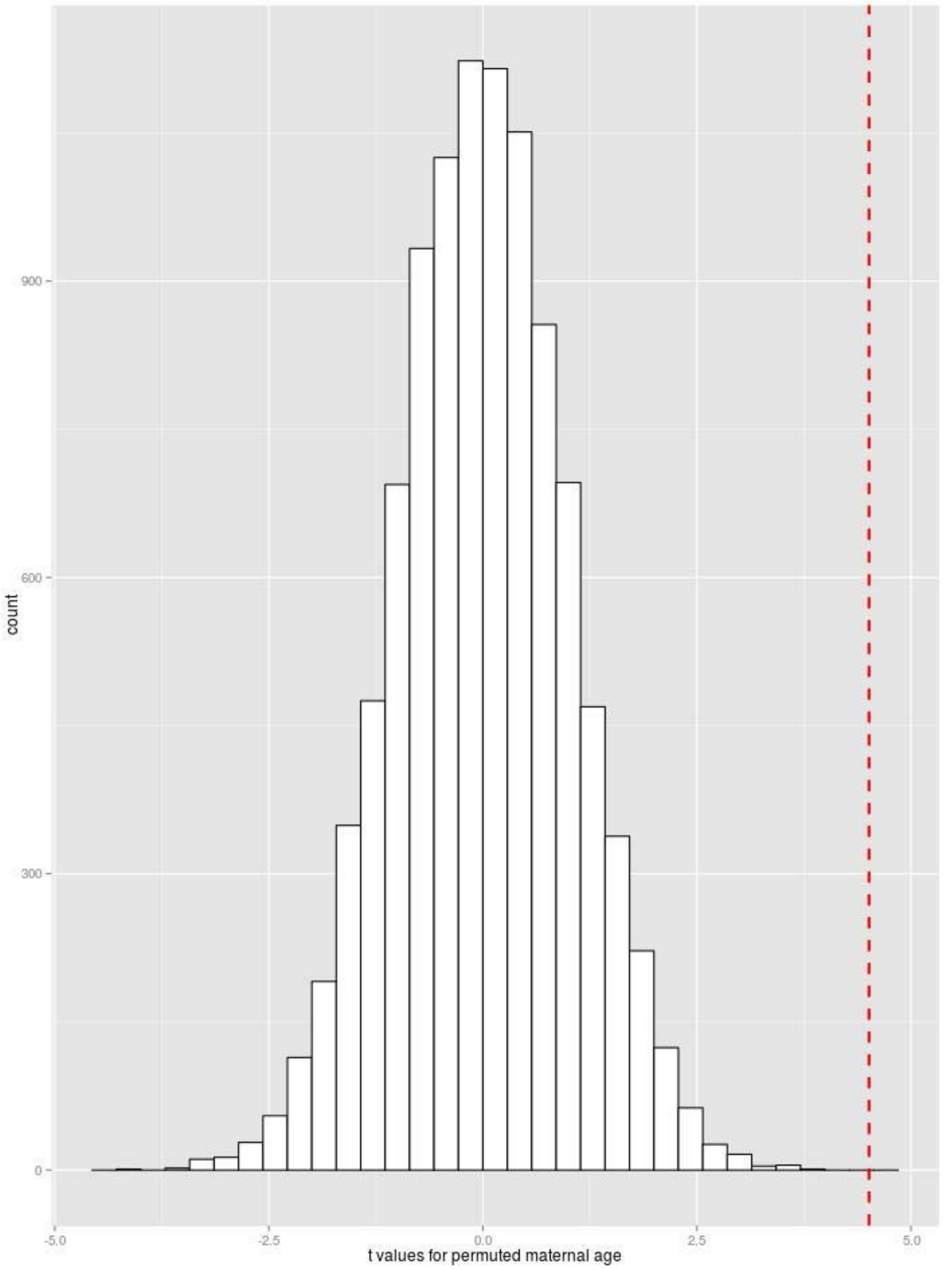

We resampled the difference between father's and mother's age and regenerated a new set of mother's age for multiple linear regression, using the number of DNMs as the response variable and the observed father's age and resampled mother's age as the explanatory variables. The procedure was repeated 10,000 times and the histogram of the T values for the resampled mother's age is plotted here. The dotted red line is the observed T value (4.56) for the original mother's age and *P* for observing this value is $< 1 \times 10^{-4}$.

Supplementary Table 1. Multiple linear fit of the total number of DNMs on parents' ages after each filtering step.

Filter	*P*(father)	*P*(mother)	R^2	total number of DNMs
Initial set	5.15×10^{-21}	2.51×10^{-4}	0.31	41730
Remove sites with mappability < 1	5.33×10^{-28}	2.17×10^{-4}	0.37	32092
Remove nearby Snps	7.08×10^{-28}	2.36×10^{-4}	0.37	31687
Remove common Variants	9.42×10^{-28}	2.37×10^{-4}	0.37	31649
Remove SegDup regions	6.00×10^{-28}	2.27×10^{-4}	0.37	31349
Remove tandem repeats regions	3.75×10^{-25}	6.05×10^{-5}	0.35	28944
Remove sites with 3rd nucleotide	5.12×10^{-25}	2.86×10^{-5}	0.36	28230

P values and R^2 of the linear fit on the number of DNMs versus father's and mother's ages after each filtering step. The general trend is that the effect of father's and mother's ages on the total number of DNMs become more significant after each filtering step.

Supplementary Table 2. Multiple linear regressions on the number of DNMs versus parents' ages on younger and older parents.

We split the dataset on total number of DNMs into two by median father's age and fitted multiple linear regression with parents' age as predictor (Tables **a** and **b**). Similarly, multiple linear regressions were performed on younger and older mothers (Tables **c** and **d**).

Model	β	S.E.	t	Pr(>t)
(constant)	6.43	3.38	1.90	0.06
father's age	0.71	0.14	4.96	1.13×10^{-6}
mother's age	0.29	0.12	2.46	0.01

(a) Linear regression model of father's and mother's age on the total number of DNM sites in the autosomes in younger fathers. R^2=0.18.

Model	β	S.E.	t	Pr(>t)
(constant)	3.32	3.95	0.84	0.40
father's age	0.67	0.10	6.54	2.22×10^{-10}
mother's age	0.41	0.11	3.77	1.95×10^{-4}

(b) Linear regression model of father's and mother's age on the total number of DNM sites in the autosomes in older fathers. R^2=0.23.

Model	β	S.E.	t	Pr(>t)
(constant)	6.63	3.36	1.98	0.05
father's age	0.68	0.09	7.30	2.05×10^{-12}
mother's age	0.31	0.14	2.29	0.02

(c) Linear regression model of father's and mother's age on the total number of DNM sites in the autosomes in younger mothers. R^2=0.22.

Model	β	S.E.	t	Pr(>t)
(constant)	-1.06	5.01	-0.21	0.83
father's age	0.59	0.09	6.72	$7.73\times10\text{-}^{11}$
mother's age	0.61	0.17	3.64	3.20×10^{-4}

(d) Linear regression model of father's and mother's age on the total number of DNM sites in the autosomes in older mothers. R^2=0.18.

Supplementary Table 3. Multiple linear regression models considering the effect of parents' age on the total number of DNMs with software versions as covariates, before and after filtering.

Model	β	S.E.	t	Pr(>t)	GVIF
(Constant)	14.78	5.42	2.72	6.60×10^{-3}	
father's age	0.99	0.09	10.85	$<2.00\times10^{-16}$	2.04
mother's age	0.39	0.11	3.47	5.56×10^{-4}	2.04
software Version 2.0.1	-4.38	4.99	-0.88	0.38	1.01
software Version 2.0.2	-2.82	4.69	-0.60	0.55	1.01
software Version 2.0.3	22.37	5.09	4.40	1.26×10^{-5}	1.01
software Version 2.0.4	-3.22	4.67	-0.69	0.49	1.01

(a) Unfiltered data: assembly software version 2.0.3 is significant ($P = 1.26 \times 10^{-5}$).

Model	β	S.E.	t	Pr(>t)	GVIF
(constant)	4.99	3.74	1.33	0.18	
father's age	0.64	0.06	10.10	$<2.00\times10^{-16}$	2.04
mother's age	0.38	0.08	4.91	1.11×10^{-6}	2.04
software Version 2.0.1	-0.52	3.44	-0.15	0.88	1.01
software Version 2.0.2	1.27	3.23	0.39	0.70	1.01
software Version 2.0.3	3.62	3.51	1.03	0.30	1.01
software Version 2.0.4	0.27	3.22	0.08	0.93	1.01

(b) Filtered data: assembly software version 2.0.3 is not significant ($P = 0.30$).

Supplementary Table 4. Multiple linear regression models considering parents' age on the total number of DNMs with fully called fraction of VQHIGH sites and software versions as covariates, before and after filtering.

Model	β	S.E.	t	Pr(>t)	GVIF
(constant)	248.82	104.96	2.37	0.018	
father's age	0.99	0.09	10.88	$<2.00\times10^{-16}$	2.04
mother's age	0.38	0.11	3.43	6.48×10^{-4}	2.04
software Version 2.0.1	-4.29	4.98	-0.86	0.39	1.13
software Version 2.0.2	-2.82	4.68	-0.60	0.55	1.13
software Version 2.0.3	21.64	5.09	4.26	2.34×10^{-5}	1.13
software Version 2.0.4	-3.78	4.67	-0.81	0.42	1.13
fully called fraction with VQHIGH	-241.64	108.23	-2.23	0.03	1.11

(a) Unfiltered data: both assembly software version 2.0.3 and fully called fraction with VQHIGH were significant ($P = 2.34 \times 10^{-5}$ and $P = 0.03$).

Model	β	S.E.	t	Pr(>t)	GVIF
(constant)	-56.20	72.67	-0.77	0.44	
father's age	0.64	0.06	10.10	$<2.00\times10^{-16}$	2.04
mother's age	0.38	0.08	4.93	1.03×10^{-6}	2.04
software Version 2.0.1	-0.54	3.44	-0.16	0.87	1.12
software Version 2.0.2	1.27	3.23	0.39	0.69	1.12
software Version 2.0.3	3.80	3.51	1.08	0.28	1.12
software Version 2.0.4	0.42	3.23	0.13	0.90	1.12
fully called fraction with VQHIGH	63.17	74.92	0.84	0.40	1.12

(b) Filtered data: none of the covariates are significant after accounting for parents' ages.

Supplementary Table 5. Multiple linear regression models considering parents' age on the total number of DNMs with gross mapping yield and software versions as covariates, before and after filtering.

Model	β	S.E.	t	Pr(>t)	GVIF
(Constant)	21.06	7.08	2.98	3.03×10^{-3}	
father's age	0.99	0.09	10.84	$<2.00\times10^{-16}$	2.04
mother's age	0.39	0.11	3.47	5.67×10^{-4}	2.03
software Version 2.0.1	-4.66	4.99	-0.93	0.35	1.01
software Version 2.0.2	-3.07	4.69	-0.65	0.51	1.01
software Version 2.0.3	22.01	5.09	4.32	1.75×10^{-5}	1.01
software Version 2.0.4	-4.33	4.74	-0.91	0.36	1.01
genome coverage gross mapping yield	-0.03	0.02	-1.38	0.17	1.06

(a) Unfiltered data: Assembly software version 2.0.3 is significant ($P = 1.75\times10^{-5}$), but not the gross mapping yield ($P = 0.17$).

Model	β	S.E.	t	Pr(>t)	GVIF
(constant)	2.71	4.89	0.56	0.58	
father's age	0.64	0.06	10.10	$<2.00\times10^{-16}$	2.05
mother's age	0.38	0.08	4.92	1.10×10^{-6}	2.04
software Version 2.0.1	-0.42	3.44	-0.12	0.90	1.67
software Version 2.0.2	1.36	3.24	0.42	0.68	1.67
software Version 2.0.3	3.74	3.51	1.07	0.29	1.67
software Version 2.0.4	0.67	3.27	0.21	0.84	1.67
genome coverage gross mapping yield	0.01	0.02	0.72	0.47	1.65

(b) Filtered data: none of the covariates are significant in the filtered data after accounting for parents' ages.

Supplementary Table 6. Sanger validation statistics on the CGI data only set and the Illumina data only set.

set	number of sites sequenced	validated	not in proband	found in parent(s)
CGI unique set	39	27	7	5
monozygotic twin set	12	11	1	0

CGI unique set (row 1) contains random DNMs selected from the CGI only calls from comparing calls from CGI custom pipeline with the two pipelines with Illumina data. Monozygotic twin set (row 2) refers to a family quartet (parents and their two monozygotic twins) that were sequenced by CGI and Illumina. In this set, 12 sites that were called with both pipelines with Illumina data, but not with CGI data, were successfully Sanger sequenced.

Supplementary Table 7. Germline mutation rates across variant types.

		CpG	Non-CpG
Transition	A->G	-	3790
	G->A	1753	3621
	C->T	1777	3500
	T->C	-	3846
	Total	3530 (7.62×10^{-8})	14757 (5.3×10^{-9})
Transversion	A->C	-	988
	C->A	112	1181
	A->T	-	810
	T->A	-	787
	G->C	78	1186
	C->G	82	1186
	G->T	92	1218
	T->G	-	932
	Total	364 (7.86×10^{-9})	8288 (3.27×10^{-9})
Summary	Ts/Tv	9.70	1.95
	S->W/W->S	-	1.44
	Total	3894 (1.98×10^{-7})	23045 (1.07×10^{-8})

The mutation rate per base per generation is shown in parentheses before correcting for the estimated false positive and false negative rate. The S→W rate is defined as total number of mutations from strong (G:C) basepairs (S) to weak (A:T) basepairs (W) per generation per effective basepairs; and vice versa for the W→S rate. The overall (CpG and nonCpG sites) S→W rate/W→S rate ratio is 1.69.

Supplementary Table 8. *P* values of the linear fit on number of DNMs versus father's and mother's ages after removing nearby SNV clusters.

	P(father)	*P*(mother)	total number of DNMs
0	1.58×10^{-22}	6.99×10^{-6}	2.72×10^{4}
10	1.30×10^{-22}	8.88×10^{-6}	2.71×10^{4}
100	1.42×10^{-22}	1.17×10^{-5}	2.71×10^{4}
1000	3.47×10^{-22}	7.61×10^{-6}	2.69×10^{4}
10^{4}	1.09×10^{-22}	2.80×10^{-5}	2.67×10^{4}
10^{5}	2.97×10^{-22}	2.39×10^{-5}	2.65×10^{4}
10^{6}	1.93×10^{-19}	1.46×10^{-5}	2.57×10^{4}
10^{7}	6.53×10^{-10}	2.85×10^{-3}	1.96×10^{4}
10^{8}	1.36×10^{-5}	1.00	4.66×10^{4}

We evaluate the effect on the significance of the estimates in the multiple linear regressions fit after removing clusters of SNVs. Both parental ages remain significant at 0.05 level after removing DNMs that are within 10^{6} bases of each other.

Supplementary Table 9. *P* values and R^2 of the linear fit on the number of DNMs versus father's and mother's ages after removing DNMs near the predicted tandem repeats regions.

	P(father)	*P*(mother)	R^2	total number of DNMs
0	3.50×10^{-22}	7.58×10^{-6}	0.35	2.69×10^{4}
10	4.52×10^{-22}	7.43×10^{-6}	0.35	2.68×10^{4}
100	1.53×10^{-21}	7.43×10^{-6}	0.34	2.58×10^{4}
1000	6.64×10^{-18}	1.12×10^{-4}	0.29	1.74×10^{4}
10000	0.98	9.33×10^{-2}	1.49×10^{-2}	507

We evaluate the effect on the significance of the estimates in the multiple linear regression fit after removing SNVs that are within 1,10, 100,...10,000 bps from the edge of any predicted tandem repeats region. Both paternal and maternal age effects remain significant after removing SNVs 10,000 bps from a tandem repeats region.

Supplementary Table 10. *P* values and R^2 of the linear fit on the number of DNMs versus father's and mother's ages after filtering DNMs at each read depth.

	father	mother	R^2	Total number of DNMs
10	3.63×10^{-22}	7.77×10^{-6}	0.35	2.69×10^{4}
15	7.21×10^{-22}	1.03×10^{-5}	0.34	2.65×10^{4}
20	6.19×10^{-22}	4.87×10^{-5}	0.33	2.50×10^{4}
25	4.91×10^{-21}	1.41×10^{-3}	0.30	2.29×10^{4}
30	1.71×10^{-18}	2.45×10^{-2}	0.25	1.97×10^{4}

We evaluate the effect on the significance of the estimates in the multiple linear regressions fit after filtering SNVs at read depths 10, 15, 20, 25 and 30. Both paternal and maternal age effects remain significant at each step. R^2 is the highest when a cutoff of 10 is used.

Supplementary Reference

1. Conrad, D. F. *et al.* Variation in genome-wide mutation rates within and between human families. *Nat. Genet.* **43,** 712–714 (2011).
